# In-Situ Biofloc Affects the Core Prokaryotes Community Composition in Gut and Enhances Growth of Nile Tilapia (*Oreochromis niloticus*)

**DOI:** 10.1007/s00248-021-01880-y

**Published:** 2021-10-05

**Authors:** Yale Deng, Klaudyna Borewicz, Joost van Loo, Marko Zabala Olabarrieta, Fotini Kokou, Detmer Sipkema, Marc C. J. Verdegem

**Affiliations:** 1 Aquaculture and Fisheries Group, Wageningen University and Research, Wageningen, The Netherlands; 2 Laboratory of Microbiology, Wageningen University and Research, Wageningen, The Netherlands; 3 Trouw Nutrition R&D, 3811 MH Amersfoort, The Netherlands

**Keywords:** Biofloc system, Ex-situ biofloc, Growth performance, Microbial community, Gut microbiota

## Abstract

Biofloc technology is commonly applied in intensive tilapia (*Oreochromis niloticus*) culture to maintain water quality, supply the fish with extra protein, and improve fish growth. However, the effect of dietary supplementation of processed biofloc on the gut prokaryotic (bacteria and archaea) community composition of tilapia is not well understood. In this study one recirculating aquaculture system was used to test how biofloc, including in-situ biofloc, dietary supplementation of ex-situ live or dead biofloc, influence fish gut prokaryotic community composition and growth performance in comparison to a biofloc-free control treatment. A core gut prokaryotic community was identified among all treatments by analyzing the temporal variations in gut prokaryotes. In-situ produced biofloc significantly increased the prokaryotic diversity in the gut by reducing the relative abundance of dominant *Cetobacterium* and increasing the relative abundance of potentially beneficial bacteria. The in-situ biofloc delivered a unique prokaryotic community in fish gut, while dietary supplementation of tilapias with 5% and 10% processed biofloc (live or dead) only changed the relative abundance of minor prokaryotic taxa outside the gut core microbiota. The modulatory effect of in-situ biofloc on tilapia gut microbiota was associated with the distinct microbial community in the biofloc water and undisturbed biofloc. The growth-promoting effect on tilapia was only detected in the in-situ biofloc treatment, while dietary supplementation of processed biofloc had no effect on fish growth performance as compared to the control treatment.

**Supplementary Information:**

The online version contains supplementary material available at 10.1007/s00248-021-01880-y.

## Introduction

Biofloc is an aggregation of microorganisms—including bacteria, microalgae, fungi and zooplankton—and other organic particles suspended in the water column [1]. In aquaculture, the formation of biofloc is stimulated by increasing the organic carbon input and aerating the culture tank, resulting in the production of microbial biomass that could serve as natural food [2]. The ability to immobilize inorganic nitrogen waste into microbial biomass makes biofloc technology a sustainable method to improve water quality and to increase the nutrient recovery from fish feed into harvested biomass [3]. For instance, the herbivorous-omnivorous Nile tilapia (*Oreochromis niloticus*) is known to eat biofloc [4], which results in a better growth than in recirculating aquaculture systems (RAS) without exhibiting adverse welfare effects [5]. Moreover, Nile tilapia grown in biofloc systems showed higher digestive enzyme activities, lower susceptibility to pathogens and a stronger immune response than in other culture systems [6, 7].

The growth-promoting effect of biofloc could be attributed to its nutritional value. Bacteria played an important role in the aquatic food web, for instance as a direct food source for aquaculture organisms, as an ingredient in formulated diets and the use of bacteria species as probiotics [8]. Biofloc harvested from Nile tilapia culture tanks had a similar or better proximate composition than the formulated feed fed to the system, with an amino acid composition meeting the nutritional requirement of tilapia [5, 9]. It has been reported that biofloc provide extra essential nutrients including proteins, lipids, essential fatty acids, minerals, vitamins, carotenoids and exogenous digestive enzymes that may improve the nutritional status of the fish and shrimp [10–12]. The in-situ biofloc produced in a tilapia culture system can be harvested, dried and used to replace fish meal in formulated shrimp feed (i.e. ex-situ biofloc), resulting in faster growth than the biofloc-free control diet [13, 14]. Therefore, biofloc is a valuable feed additive that contributes to the re-use of waste nutrients for sustainable aquaculture. However, the dietary role of biofloc in the growth performance of culture species needs further investigation.

Recently, biofloc has been applied as a strategy for disease management due to its probiotic effect on animal health [15]. The beneficial bacteria and its bioactive compounds, such as polyhydroxy butyrate, present in biofloc could increase the immune response and protect shrimp or fish against bacterial infections [6, 16, 17]. The high concentration of organic matter and its associated load of microorganisms in a biofloc system can influence the gut microbiota due to the constant grazing of the culture species on biofloc. Nile tilapia larvae cultured in biofloc system showed different gut microbiota composition with tilapia grown in RAS [18]. Biofloc produced with different carbon sources or feed ratios resulted in different microbial community composition in the gut of tilapia [9, 19, 20]. Gut microbiota played an important role in nutrient digestibility and immune response, thus influencing the growth and health of aquatic animals [21–23]. However, how in-situ biofloc influence the gut microbiota composition in Nile tilapia and whether the dietary supplementation of ex-situ biofloc can change the gut microbiota and growth of tilapia remains unknown.

In aquaculture, a probiotic was defined as “a live microbial adjunct which has a beneficial effect on the host by modifying the host-associated or ambient microbial community, by ensuring improved use of the feed or enhancing its nutritional value, by enhancing the host response towards disease” [24]. Therefore, to distinguish between the nutritional value and probiotic effect of biofloc on fish, in-situ biofloc were harvested and incorporated into fish feed as live biofloc or γ-radiated dead biofloc. Giatsis et al. [25] showed large variations existing in the water and fish gut microbial community of replicate culture systems. Therefore, in this study one recirculating system was used for all the treatments to minimize system effects on water microbial community composition in the rearing tanks. In the recirculating system, live biofloc feed (LF), dead biofloc feed (DF) and biofloc-free feed (Ctrl) were fed to tilapia. In addition, the biofloc-free feed was also fed to tilapia swimming in tanks with in-situ biofloc (LW). The aim of this study was to test how in-situ biofloc (LW) and dietary supplementation of ex-situ biofloc (DF and LF) influence the gut microbiota development and the growth performance of tilapia as compared with the Ctrl treatment.

## Materials and Methods

### Experimental Setup and Animal Accommodation

**Fig. 1:**
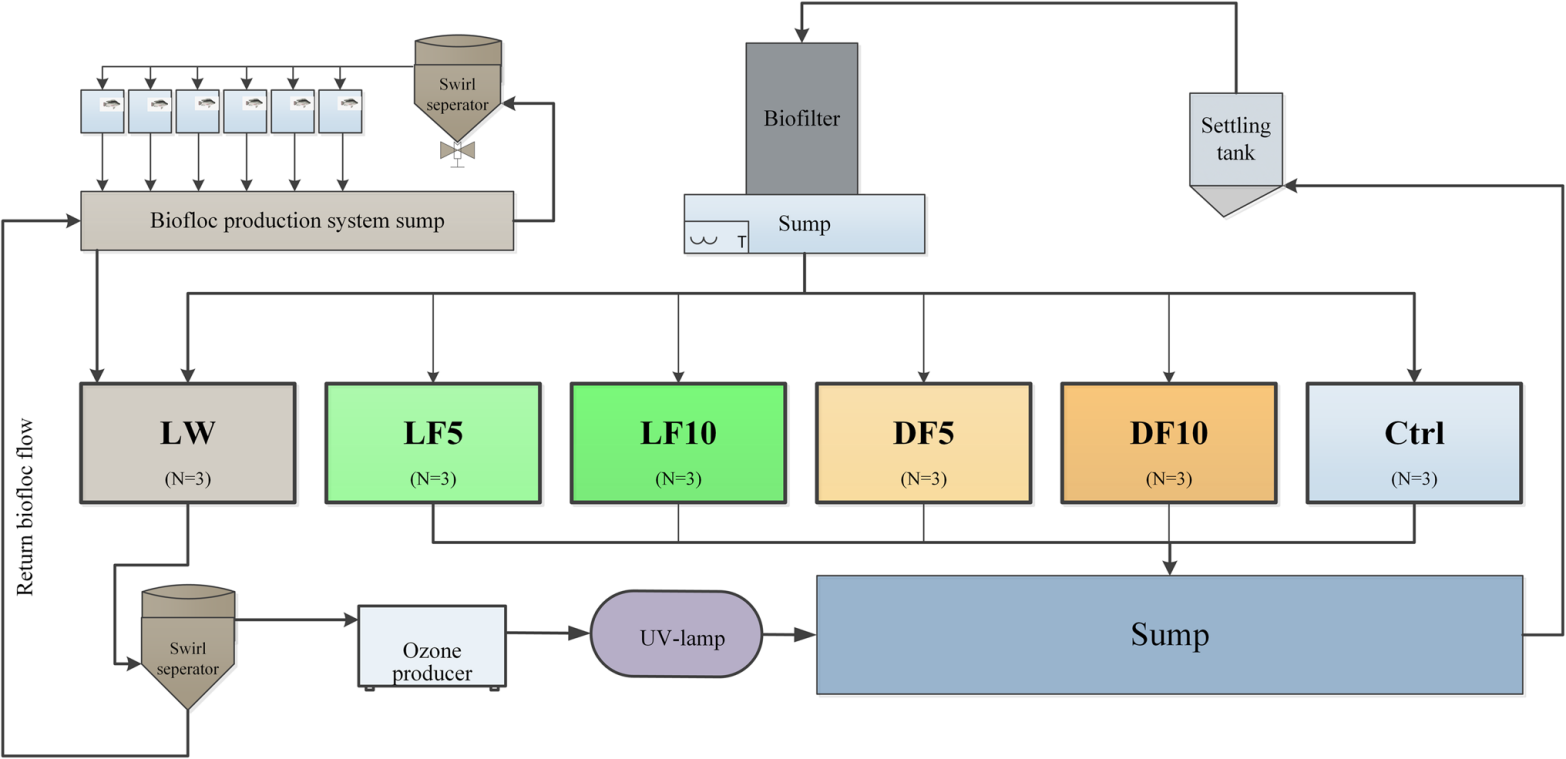
Experimental setup and treatment groups. Experimental fish were stocked in six treatment groups with each group containing three replicates (*N* = 3). LW treatment received in-situ biofloc water originating from a biofloc production system and was fed with control diet. LF and DF treatment received a diet containing the same feed ingredients as control diet, plus ex-situ live biofloc or dead biofloc, respectively, at 5% (LF5, DF5) or 10% (LF10, DF10) on dry weight basis. Ctrl treatment received control diet without biofloc

This experiment was carried out between October, 2015 and November, 2015. Six treatments were randomly divided over eighteen 30-L tanks (3 replicates per treatment) that were part of one recirculating aquaculture system (Fig. 1). The live water (LW) treatment received biofloc water from a biofloc production system in which six tilapia fish tanks was placed for biofloc production. Biofloc water was also collected daily from a swirl separator (Fig. 1, swirl separator top-left) and passed through a paper filter. The collected biofloc on filters was semi-dried at room temperature in the lab until 5% moisture. The unprocessed biofloc contained on a dry matter basis (g/kg) 513 crude protein, 317 carbohydrates and 110 ash. In this study, the control diet was prepared by Research Diet Services BV in the Netherlands, using mainly wheat, maize, fish meal and soya bean meal as raw ingredients. The feed contained on a dry matter basis 36.2% crude protein, 6.2% crude fat, 7.2% crude ash, 23.9% starch and 1.05% total phosphorous. The control diet was crumbled and mixed with the semi-dried biofloc, on a dry matter basis at a dose of 5 or 10% of the control feed amount. Half of the feed obtained in this way is referred to as live biofloc feed (LF5 at 5% addition and LF10 at 10% addition). The other half of the feed was γ-radiated (Synergy Health, Ede, the Netherlands) at the dose of 8000 Gy (Gy) to kill microbiota, which is referred as dead biofloc feed (DF5 at 5% addition and DF10 at 10% addition). Both LW and Ctrl treatments received control diet which was also crumbled into a dough and processed into pellets, following the same process as for the LF and DF treatments. The effluent of LW tanks was led to a swirl separator (Fig. 1, swirl separator bottom-left) to return the biofloc back to production system and the overflow was passed through an ozone treatment unit and a UV-lamp to oxidize and inactive the bacteria remaining in biofloc water. A sump (Fig. 1, bottom-right) was placed to receive the effluents from all the treatment tanks and maintain the water temperature at 28 °C with a heater. From the sump, the water was pumped to a settling tank for solids removal and subsequently over a trickling biofilter for ammonium removal before returning to each treatment tank.

In each tank, 33 Nile tilapia (Til-Aqua, Velden, the Netherlands) were stocked at the start of the experiment with an average body weight of 0.58 g. The fish were fed using a belt feeder from 10 a.m. over a period of 10 h daily at the ratio of 16 g per kg metabolic weight (16 g kg^−0.8^ day^−1^). The diet applied in this experiment was a commercial diet that was sterilized by γ-radiation with a dose of 8000 Gy before use in all treatments. All experimental treatments were randomly assigned to the tanks to exclude the potential influence of location-associated factors.

### Samples Collection from Gut, Water, Flocs and Feed

Fish gut, tank water, biofloc and feed samples were collected on days 0, 26 and 49 according to a previously described protocol [25]. Ten fish were sampled on day 0 for the analysis of the initial gut microbiota, and 5 fish per tank were randomly sampled at later timepoints for the intestinal microbiota analysis on days 26 and 49. Fish were euthanized with 0.6 g L^−1^ Tricaine Methanesulfonate (TMS, Crescent Research Chemicals, Phoenix, Arizona, USA), then rinsed with 70% ethanol and sterile water before dissecting out the gut. Moreover, 100 mL of water from each tank was collected in three replicates and filtered through 0.45 and 0.22 μm membrane filters (Millipore—Isopore), the two filters were pooled together for DNA extraction. In addition, 100 mL water was also sampled in five replicates from the biofloc production system sump and filtered over a paper filter to collect biofloc. Finally, 2 g samples of feed prepared for LF5, LF10, DF5, DF10 and LW/Ctrl (control diet) were collected in three replicates. All samples were flash frozen in liquid nitrogen and stored individually at − 80 °C for the prokaryotic community composition analysis. Moreover, the average individual weights of fish on days 0, 26 and 49 was measured to evaluate the fish growth performance during the experiment.

### Genomic DNA Extraction and 16S rRNA Gene Sequencing

DNA extraction from fish gut samples was conducted using the DNeasy Blood & Tissue Kit (Qiagen, Venlo, Netherlands), whereas the FastDNA SPIN kit for soil (MP Biomedicals, Ohio, USA) was used for water, feed and biofloc samples. The V4 region of 16S rRNA gene was amplified using uniquely barcoded primers 515F (5′-GTGCCAGC[AC]GCCGCGGTAA-3′) and 806R (5′-GGACTAC[ACT][ACG]GGGT[AT]TCTAAT-3′) [26]. The amplicon libraries were HiSeq sequenced (GATC-Biotech, Konstanz, Germany) and sequencing data were processed and analyzed with NG-Tax using default parameters [27]. In brief, libraries were filtered to contain only read pairs with perfectly matching barcodes that were subsequently used to separate reads by sample. Unique sequences (operational taxonomic units, OTUs) occurring above a minimum 0.1% relative abundance threshold per sample were picked, and subjected to non-reference-based chimera checking, where the parent sequence needed to be more abundant by a 0.5 ratio than the chimeric sequence. Taxonomy was assigned using the SILVA_111_SSU reference database [28]. Samples with a low number of sequencing reads (< 1500) were removed from analysis.

### Data Analysis

The fish growth performance during the experiment was assessed by weight gain (WG), and metabolic growth rate (MGR), which were calculated as previously described [29]: WG = *W*_*f*_ – *W*_*i*_, GBW = ${e}^{({\text{ln}}{\text{W}}_{\text{f}} + {\text{ln}}{W}_{i})/2}$, MGR = ${\text{WG}}*{\left(\frac{\text{GBW}}{1000}\right)}^{-0.8}/t$, where *W*_*f*_ (g) and *W*_*i*_ (*g*) are the final and initial average body weight, WG (g) is the weight gain, GBW (g) is the geometric mean body weight, and MGR (g kg^−0.8^ day^−1^) is the metabolic growth rate, *t* is the number of days. The growth performance was expressed in metabolic body weight to minimize the differences in maintenance levels between smaller and larger fish. The growth performance was compared among the six treatments by repeated measures ANOVA to analyze the interaction between treatments and growth for the periods of d0–d26 and d27–d44 using IBM SPSS statistics software.

A total of 391 experimental samples were sequenced, with a combined number of 65,383,991 sequencing reads (range 4 to 740,934 reads, median = 138,760). Four samples, including one Ctrl, two DF water and one LW feed, were removed from analyses due to the low number of reads (< 1500). Series of rarefactions were performed prior to alpha diversity analyses at cut offs between 1500 and 10,000 reads. The cutoff value was then chosen to provide maximum coverage while allowing most samples to be retained for the analyses. As a result, data were rarified at 2500 reads and four gut samples were removed because their read numbers were below this threshold. Alpha diversity indices, including Shannon, Chao1, and Phylogenetic Diversity (PD) Whole Tree, for each gut sample were calculated on genus level data using QIIME [30]. Resulting diversity scores were compared between different treatment groups using nonparametric *t*-test with Monte Carlo permutations (*n* = 999). Statistical differences in relative abundance (RA) of genus level taxa and phylotypes (OTUs) between treatments were assessed with Kruskal–Wallis test using QIIME. The UniFrac distance between all gut samples based on weighted data were compared using analysis of similarity (ANOSIM) test in QIIME. Principal coordinate analysis (PCoA) and redundancy analysis (RDA) were calculated in Canoco5 using the log transformed genus level relative abundances data. Statistical between treatments was assessed in Canoco5 under the full model using the Monte Carlo permutation test with 499 random permutations [31].

## Results

### Fish Growth Performance

At the start on d0, fish from each tank had a similar average body weight (0.58 g) for all treatments (Table 1). During d0–d26, LW treatment showed a significantly (*P* < 0.05) higher final body weight and weight gain than the other treatments except for LF10, resulting a higher metabolic growth rate than all the other treatments. No significant (*P* > 0.05) difference in weight gain and metabolic growth rate was detected between all treatments during period d26–d44. Repeated measures ANOVA analysis revealed that both treatment and time had a significant (*P* < 0.05) effect on the fish weight gain and metabolic growth rate. Moreover, significant interaction between time and treatment concurred with the observed disappearance of significant difference between treatments from period d0–d26 to period d27–d44. Overall, LW resulted in a higher weight gain and metabolic growth rate than the Ctrl, fish from LF10 treatment achieved similar growth as LW, whereas dietary supplementation of dead biofloc at 5 and 10% (DF5 and DF10) or live biofloc at 5% (LF5) had no effect on fish growth as compared with the Ctrl treatment.

**Table 1:** Growth performance of all treatments during d0–d26 and d26–d44

Time	Growth parameter	Treatment						SEM	*P* value		
		Ctrl	DF5	DF10	LF5	LF10	LW		Treatment	Time	Treatment* Time
d0–d26	Initial body weight (g)	0.57	0.58	0.58	0.56	0.62	0.57	0.02	ns	na	na
	Final body weight (g)	3.12^b^	3.05^b^	3.06^b^	3.01^b^	3.30^ab^	3.56^a^	0.09	**		
	Weight gain (g)	2.55^b^	2.46^b^	2.48^b^	2.45^b^	2.67^ab^	2.99^a^	0.07	**		
	Metabolic growth rate (g kg^−0.8^ d^−1^)	20.31^b^	19.63^b^	19.84^b^	19.85^b^	20.14^b^	22.60^a^	0.31	***		
d26–d44	Initial body weight (g)	3.12^b^	3.07^b^	3.11^b^	3.07^b^	3.33^ab^	3.56^a^	0.08	^**^	na	na
	Final body weight (g)	8.71	8.68	8.68	8.59	9.32	9.89	0.31	^#^		
	Weight gain (g)	5.59	5.61	5.58	5.53	5.99	6.33	0.24	ns		
	Metabolic growth rate (g kg^−0.8^ day^−1^)	20.8	21.09	20.81	20.86	21.16	21.23	0.45	ns		
d0–d44	Weight gain (g)	4.07^b^	4.04^b^	4.03^b^	3.99^b^	4.33^ab^	4.66^a^	0.13	^*^	^***^	ns
	Metabolic growth rate (g kg^−0.8^ day^−1^)	20.56^b^	20.36^b^	20.33^b^	20.36^b^	20.65^b^	21.92^a^	0.27	^**^	^*^	^*^

*SEM* standard error of the mean, *ns* not significant, *na* not applied

^#^*P* < 0.1, *P < 0.05, **P < 0.01, ***P < 0.001. The different superscript letters indicated the significant difference between treatments

### Gut Microbial Diversity on Day 0, 26 and 49

**Fig. 2:**
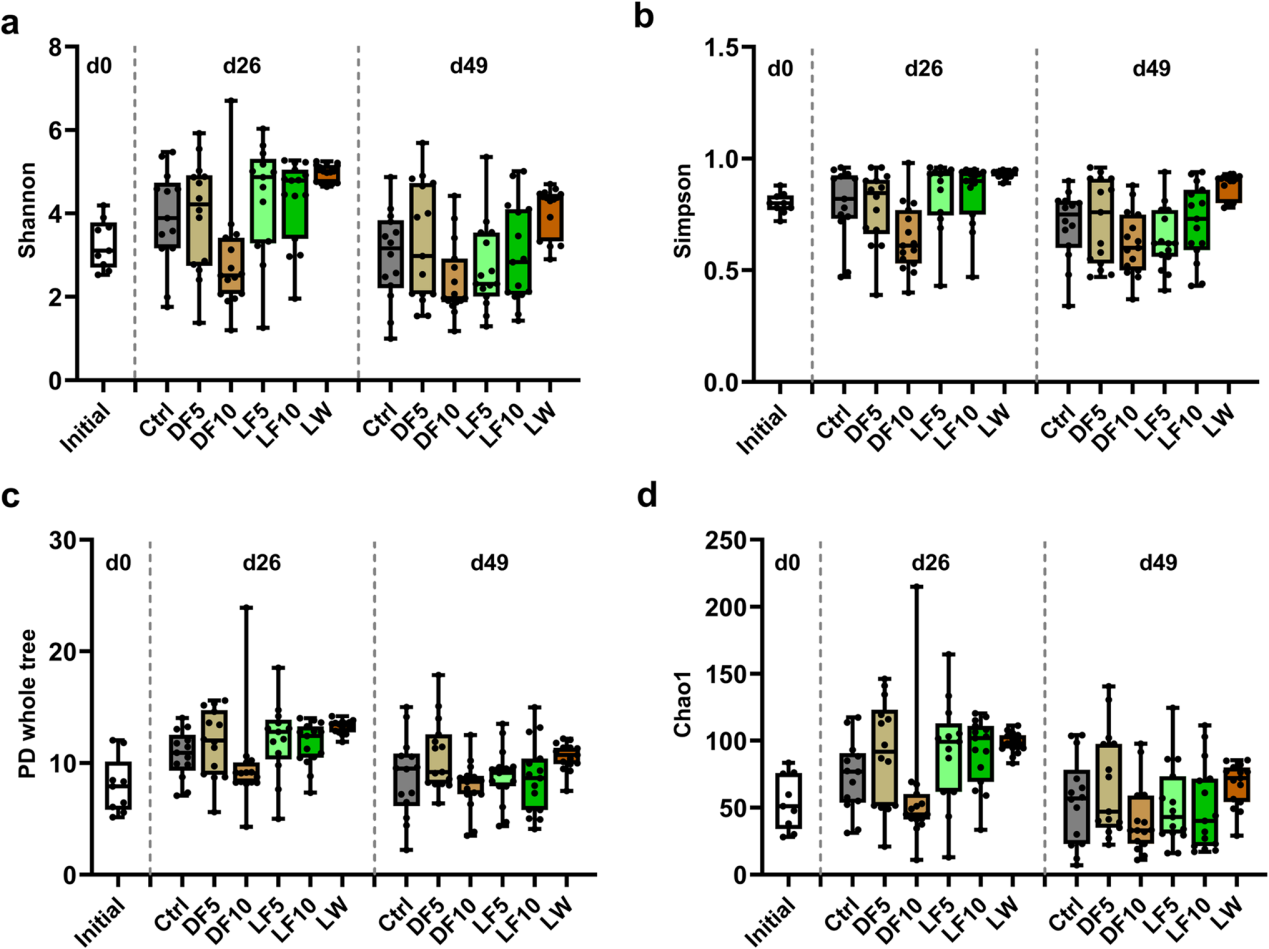
Changes in alpha diversity indexes of Nile tilapia gut prokaryotic community from six treatments at day 0, 26 and 49. **a** Shannon index, **b** Simpson index, **c** PD whole tree index, **d** Chao1 index

A total of 183 gut samples were analyzed from the six treatment groups at d0, d26 and d49, which revealed the presence of 1298 different OTUs. Among them, 611 OTUs were detected in two or more samples, which were used for further analysis. The alpha diversity indexes are shown in Fig. 2 and the statistical analysis result is shown in Table S1. At both d26 and d49, LW showed the highest alpha diversity indexes and the lowest variations of the prokaryotic communities in all treatment groups. At d26, LW showed significantly (FDR < 0.05) higher diversity for the Shannon, PD whole tree and Chao1 indexes than Ctrl, while the other treatments had similar alpha diversity indexes as Ctrl. At d49, LW only showed significantly higher (FDR < 0.05) diversity than Ctrl for the Shannon index, but not for the PD whole tree and Chao1 indexes. Still, fish fed with ex-situ biofloc (LF and DF) had similar alpha diversity for all indexes as Ctrl treatment at d49. We also compared the alpha diversity shift over time, which revealed that fish receiving in-situ biofloc or ex-situ live biofloc in LW, LF5 and LF10 showed a significant (FDR < 0.05) increase from d0 to d26 and then a significant (FDR < 0.05) decrease from d26 to d49 in the Shannon, Chao1 and PD whole tree indexes. This resulted in a similar alpha diversity index between d0 and d49 for fish fed with ex-situ live biofloc while LW still showed significantly higher diversity at d49 than d0 for the Shannon and PD whole tree indexes but not in Chao1 index. On the other hand, fish from DF5 and DF10 did not show significant change in prokaryotic diversity over time.

### Gut Prokaryotic Community Distribution

**Fig. 3:**
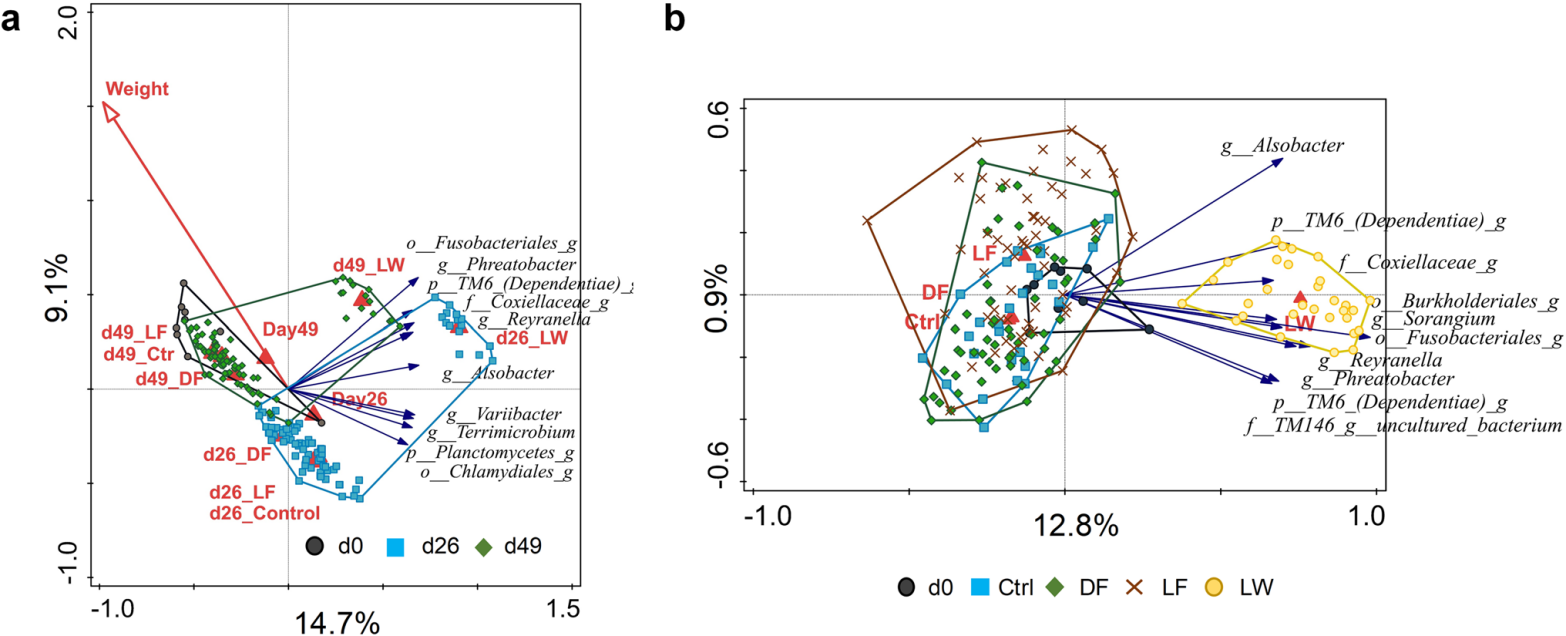
RDA displaying 10 best fitting genus level taxa to explain the variations in gut prokaryotes. **a** Samples color coded by timepoint; **b** samples color coded by treatment group with timepoint as covariate

To investigate the distribution of the intestinal prokaryotic community, RDA was performed on the relative abundance of genera using collection timepoint, treatment, fish body weight and tank number as explanatory variables (Fig. 3a). Together these variables accounted for 36.3% variation in the data, with a significant (FDR < 0.05) conditional effect of variables “d26_LW” (9.6% variation), “d49_LW” (5.9% variation), and “d26_DF” (1.2% variation). There was a clear separation of samples by collection timepoint and LW treatment. Similarly, the PCoA results also indicated the separation of samples based on collection timepoint and separation of the LW treatment from the other treatments at both d26 and d49 based on weighted UniFrac analysis (Fig. S1). To remove the effect of time, we set the variable “timepoint” as covariate and repeated the RDA analysis with treatment as explanatory variable (Fig. 3b). The result again showed a significant conditional effect of LW, and a trend effect (FDR = 0.06) of LF, with a clear separation of LW from the other treatments.

The ANOSIM test showed that all treatments resulted in unique (*P* < 0.05) gut microbiota profiles of prokaryotic community composition based on weighted Unifrac data on d26 (Table S2). On d49, only DF and LW prokaryotic community composition was significantly (*P* < 0.05) different from Ctrl when weighted UniFrac distances were compared.

### Gut Prokaryotic Community Composition

**Fig. 4:**
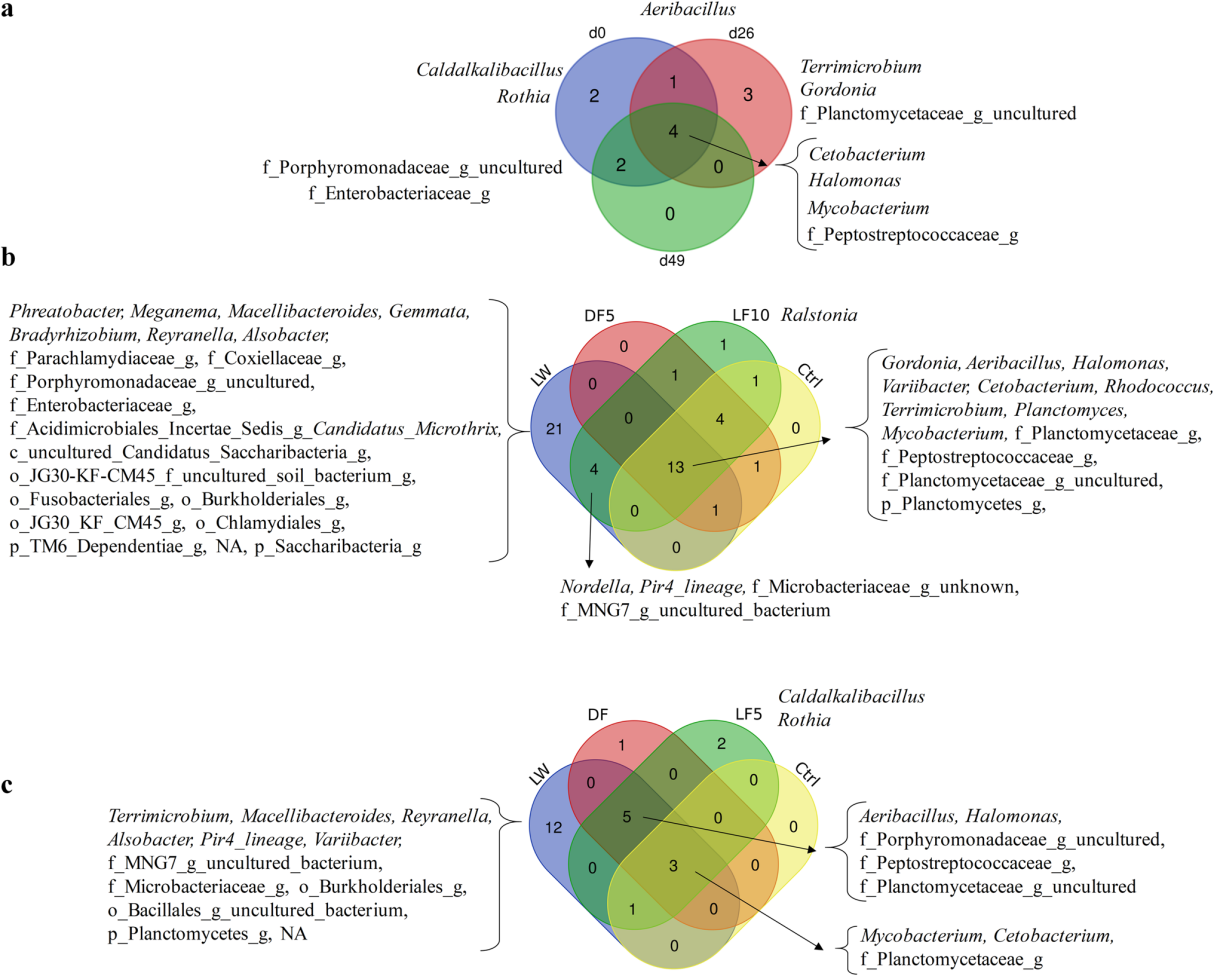
Venn diagrams showing shared genus level taxa found in more than 95% of samples **a** within three sampling timepoints, **b** on d26, and **a** on d49

In all the gut samples, a total of 487 genera was identified, of which the genera detected in more than 95% of the samples were referred to as the core genera (Fig. 4). The four core genera, namely *Cetobacterium*, *Halomonas*, *Mycobacterium* and an unidentified genus within the family *Peptostreptococcaceae,* varied slightly over each timepoint. To be noticed, the four core genera also had high average relative abundance for all the gut samples, *Cetobacterium* (average RA = 49%, range 0.8–96%, prevalence = 100%), *Mycobacterium* (average RA = 7%, range 0–26%; prevalence = 99%), an unidentified genus within the family *Peptostreptococcaceae* (average RA = 5%, range 0–43%, prevalence = 97%), and *Halomonas* (average RA = 4%, range 0–29%, prevalence = 97%). However, the number of core genera for all treatments decreased from thirteen at d26 to three at d49. The shift of core genera was consistent with the result that the prevalence of the main genera oscillated with time and between treatment groups (Fig. S2).

There was no significant difference in the relative abundance of gut genera between 5% and 10% supplementation of ex-situ biofloc in both DF and LF treatments. Therefore, the DF5 and DF10 treatments were pooled as DF, and LF5 and LF10 treatments were pooled as LF. There were 77 genus level taxa on d26, and 44 genus level taxa on d49 varied significantly (FDR < 0.05) in relative abundance between all treatments (i.e. Ctrl, LF, DF and LW). Thirty-five of these differentially abundant taxa were detected at both timepoints (Table 2, with the full taxonomic classification of the taxa provided in Table S3), indicating that the treatment effects on these taxa were relative stable over time. Among these 35 taxa, the core genera *Cetobacterium* and *Mycobacterium* were also detected with more than 95% prevalence in the gut samples.

**Table 2:**
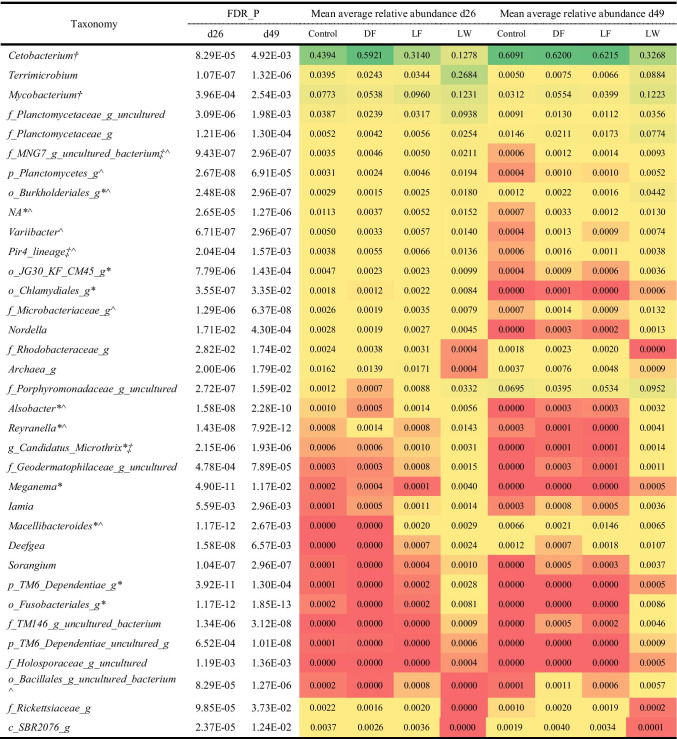
Differentially (FDR < 0.05) abundant genera taxa of intestinal prokaryotes between treatments on both d26 and d49

^†^Core taxa in d26 and d49; *core taxa in LW d26; ^^^core taxa in LW d49; ^‡^core taxa in LF + LW, core taxa means the taxa detected in more than 95% of the samples. Values are colored based on graded color scale where red indicates the lowest value, yellow indicates the percentile, and green indicates the highest value

Moreover, pairwise comparisons of relative abundances at genus level for each of the treatment indicated no significant (FDR < 0.05) differences between LF vs. Ctrl and DF vs. Ctrl on d26 and d49. However, differentially abundant taxa could be found between LW and Ctrl, including 63 taxa on d26 and 30 taxa on d49. There were 21 taxa differing between LW and Ctrl on both d26 and d49, which is shown in Table 3. The specific taxa that only significantly varied in LW vs. Ctrl but not in LW vs. LF groups were *Cetobacterium*, *Cupriavidus*, *Iamia* and unidentified genera of the family Brevinemataceae on d26, and *Turicibacter* and unidentified genera of the family Peptostreptococcaceae and the order Rhizobiales on d49 (Table S4). This finding suggested the unique and specific effect of the LW treatment on the gut microbial community in Nile tilapia.

**Table 3:**
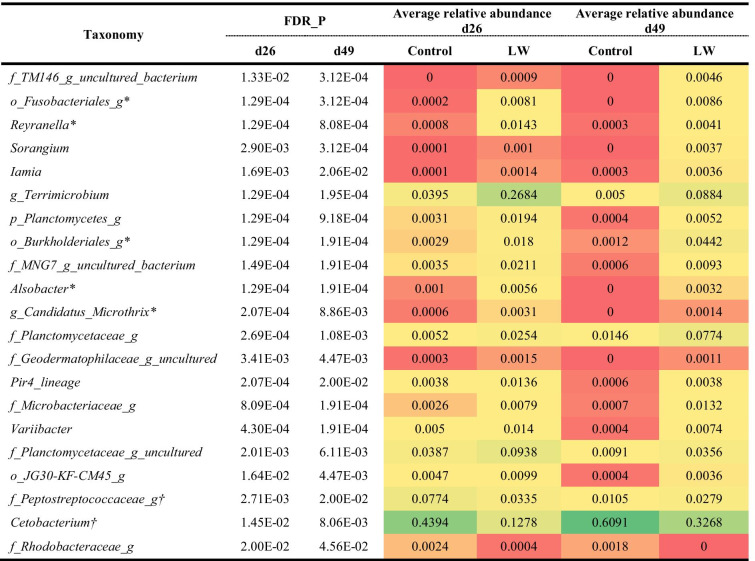
Differentially (FDR < 0.05) abundant genera taxa in pairwise comparisons of intestinal prokaryotes between LW and Ctrl on both d26 and d49

^†^core taxa in all treatments on d26 and d49; *core taxa in LW on d26 and d49; core taxa means the taxa detected in more than 95% of the samples. Values are colored based on graded color scale where red indicates the lowest value, yellow indicates the percentile, and green indicates the highest value

### Water and Feed Microbial Community

**Fig. 5:**
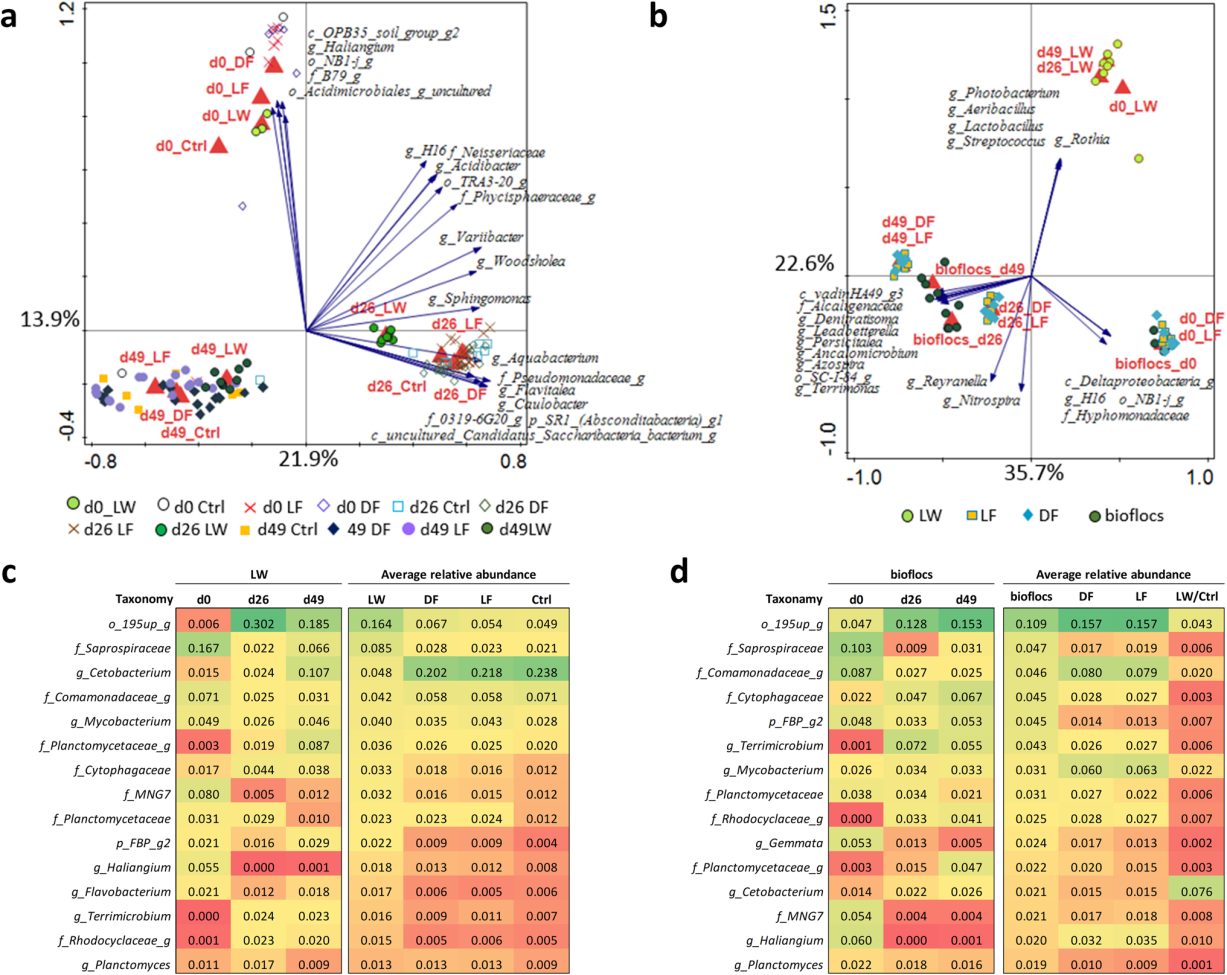
Filtered tank water and feed prokaryotic community composition. RDA plots of **a** filtered tank water and **b** feed samples. Heatmap showing the relative abundance of top 15 abundant genera in **c** LW water and other water samples and in **d** bioflocs and other feed samples. Bioflocs were harvested from the biofloc production tank and used to formulate DF and LF feed by mixing with control diet. LW/Ctrl received the control diet

The harvested bioflocs, feed samples and filtered tank water showed a distinct prokaryotic community composition when compared with the gut samples according to PCoA plot (Fig. S3). There was a clear separation (FDR < 0.05) among water samples based on collection timepoint, and treatments were clustered within the same timepoint (Fig. 5a). The ANOSIM results showed that DF and LF had similar prokaryotic community in the water with Ctrl on the three timepoints (Table S5a). On the other hand, LW exhibited a significantly different water prokaryotic community on d26 and d49 when compared with all the other treatments. The LW water was dominant with a genus belonging to the order *195up* (average RA = 16%), while the water from DF, LF and Ctrl was dominant with *Cetobacterium* (average RA = 22%) (Fig. 5c)*.* The RDA plot implied that the variations in water samples between different sampling timepoints were caused by low abundant genera instead of the dominant genera shown in Fig. 5c.

In this study, bioflocs were harvested from a biofloc production system to make the LF and DF feed by mixing with the control diet which was presented as LW/Ctrl feed in Fig. 5b. The prokaryotic community in LW/Ctrl feed were clustered together at all the three sampling timepoints and showed significant (*P* < 0.05) difference with the LF and DF feed according to ANOSIM test (Table S5b). The prokaryotic community in bioflocs changed over time and showed significant difference with the LF feed and DF feed on each timepoint. On the other hand, no significant differences were observed between the DF and LF feed at all the three timepoints. The LW/Ctrl feed was dominant with *Cetobacterium* (average RA = 7.6%) and a genus belonging to the order *195up* (average RA = 4.3%). The LF and DF feed were dominant with a genus belonging to the order *195up* (average RA = 14%), a genus belonging to family Comamonadaceae (average RA = 7.9%) and *Mycobacterium* (average RA = 6.2%). Like the water samples, the variations between feed samples were caused by low abundant genera instead of the dominant genera as shown in Fig. 5d.

Moreover, we compared the bioflocs (*n* = 10), LW filtered water (*n* = 17) and LW fish gut (*n* = 30) samples collected on both d26 and d49 (Fig. S4). There were 25 taxa that were shared between bioflocs, LW filtered water and LW gut samples. Among these taxa *Mycobacterium*, *Terrimicrobium*, *Cetobacterium* and unidentified genera within the families Planctomycetaceae and Porphyromonadaceae showed 100% prevalence in all the samples and had higher average relative abundance in the gut of Nile tilapia in the LW treatment than in the gut of fish from other treatments, which suggests that these taxa might originate from the bioflocs or LW tank water.

## Discussion

### LW Promoted Tilapia Growth, DF and LF Showed No Enhancement in Growth

In this study, fish cultured in the in-situ biofloc (LW) had a weight gain of 15% higher than the Ctrl that were not exposed to biofloc (Table 1). This enhancement in fish growth falls in the reported range that biofloc systems had 9–27% higher fish production than the flow-through system or recirculating system [6, 32, 33]. Biofloc grown in the biofloc system were shown to be an effective food source for tilapia [4, 5]. The fact that biofloc improves fish production is potentially due to the provision of extra food or the enhancement in feed digestion [1, 3]. However, the growth-promoting effect of dietary supplementation of live and dead biofloc (LF and DF) were not detected in our study. Our studies demonstrated that dietary supplementation of γ-radiated biofloc at 5% or 10% weight base (DF5 and DF10) had no effect on tilapia growth, while the dietary supplementation of live biofloc at 10% (LF10) showed similar growth as in the Ctrl and LW treatments. In this study, an increasing trend in tilapia growth was observed with the increased dose of ex-situ live biofloc in the feed, which was not observed in the ex-situ dead biofloc feed. This suggested that the processing of biofloc reduced its nutritional quality to Nile tilapia, which was in line with previous study that tilapia in the in-situ biofloc system grown better than dietary supplementation of biofloc [34]. The micronutrients (e.g. vitamins and free amino acids) or bioactive compounds (e.g. enzymes and carotenoids) in biofloc could be degraded or oxidized differently because of the heat, grinding, air or light submitted during the processing [11]. In our processing, the collection of biofloc from a swirl separator followed by filtration over a paper and subsequent dewatering, may result in the loss of active ingredients or bacteria in the in-situ biofloc that can enhance tilapia growth as observed in the LW treatment. Therefore, the growth-promoting effect of in-situ biofloc on Nile tilapia was confirmed in this study, while processing and dietary supplementation of biofloc may reduce its nutritional quality.

### LW Changed the Gut Microbiota Diversity and Distribution

In addition to the growth-promoting effect of in-situ biofloc, some potential probiotic effects of biofloc were reported on cultured animals, such as modulation on gut microbiota, inhibition of pathogenic microorganisms and improvement of immune response [15, 24, 35]. Many studies have demonstrated the in-situ biofloc were associated with the shift of gut microbiota of shrimp [36–40]. However, studies on the effect of in-situ biofloc and dietary supplementation of ex-situ biofloc on the gut microbiota of tilapia are still rare. LW significantly increased the microbial diversity and richness in the gut of tilapia when compared with Ctrl (Fig. 2). Higher Shannon index and Chao 1 index were also observed in the gut of *Litopenaeus vannamei* reared in biofloc system than in the clear water system [41]. The introduction of live bacteria from biofloc to fish through water contact or flocs ingestion could act as bacterial source for colonization and thus increase the bacterial diversity and richness in the gut [19, 42]. Improvement in microbial diversity is not always beneficial to the host, however, from the ecological point of view, higher taxonomic diversity is associated with higher functional redundancy and higher stability of the gut microbiota [43]. A more diverse gut microbiota was assumed to be beneficial to the fish, since higher growth performance and lower individual variations of the LW fish were observed in this study. The difference in gut microbial diversity between LW and Ctrl was attributed mostly to unrelated taxa on d26 since different PD whole tree index was observed [44]. On d46, the difference in microbial diversity between LW and Ctrl was caused by phylogenetically related taxa since the difference in PD whole tree index disappeared. In contrast, no significant differences were observed between DF vs Ctrl and LF vs Ctrl in the fish gut microbiota diversity, which implied that dietary supplementation of live or dead biofloc cannot mimic the microbial composition in the in-situ biofloc.

Furthermore, tilapia cultured in the in-situ biofloc (LW) exhibited a distinct gut microbiota composition when compared with other treatments while dietary supplementation of ex-situ biofloc (DF and LF) had similar gut microbiota composition as the Ctrl (Fig. 3). The gut microbiota development is thought relating with the changes of gut structure and functionality and the variations in water and feed microbial communities [23, 25, 45]. Temporal change in the fish gut microbiota composition was commonly observed in aquaculture [46, 47]. The temporal changes in the gut microbiota might be associated with the fact that the prokaryotic community in the rearing water (Fig. 5a), biofloc and feed (Fig. 5b) shifted over time. According to Giatsis et al.[25], the water samples collected from different tanks in one RAS had no difference in bacterial community. This explained that the Ctrl, DF and LF treatments had similar microbial community in the tank water from the same sampling timepoint (Table S5). However, due to the continuous influent from biofloc production system to the LW tanks, LW showed difference in water microbial community composition with other treatments. Moreover, the prokaryotic community in the feed for DF and LF were always different from the live biofloc (Fig. 5), implying that processing of biofloc changed the microbial composition in the in-situ biofloc. Therefore, the observed differences in the gut microbiota of tilapia in LW are associated with the unique microbial community created in both the in-situ flocs and the rearing water. However, this modulatory effect on gut microbiota was not observed through dietary supplementation of biofloc which might change the microbial composition in the processing of biofloc.

### Taxa Associated with the LW Treatment

According to a standard procedure, the unique OTUs detected only in one sample was excluded from analysis [48]. With the temporal changes in fish gut microbiota development, four core genera showed more than 95% prevalence in all the gut samples (Fig. 4). Species belong to *Cetobacterium* and Peptostreptococcaceae were widely detected in the gut of Nile tilapia with high abundance [9, 18, 49–52]. The dietary treatment of in-situ or ex-situ biofloc changed the relative abundance of core gut microbiota of tilapia (Table 2). Examining the relative abundance of genera showed that LW changed the core taxa when compared with Ctrl treatment (Table 3). The relative abundance of *Cetobacterium* in fish gut was lower in LW (RA = 12.8% on d26, 32.7% on d49) than the Ctrl (RA = 43.9% on d26, 60.9% on d49). This could be explained by the high abundance of *Cetobacterium* in the water from the Ctrl (RA = 24.2%), DF (RA = 25.6%) and LF (RA = 23.6%), while only 6.0% was detected in the LW water. The effect of LW on the relative abundance of Peptostreptococcaceae was less clear, since Peptostreptococcaceae was lower on d26, but higher on d49 than the Ctrl. In contrast, *Terrimicrobium* showed a higher relative abundance in LW (RA = 26.8% on d26, 8.8% on d49) when compared with Ctrl (RA = 4.0% on d26, 0.5% on d49). This could be explained by the relatively high abundance of *Terrimicrobium* in the biofloc and LW water (Fig. 5). Besides, those bacteria were detected with high prevalence and relative abundance in bioflocs, LW filtered water and LW fish gut (Fig. S4). In addition, 12 microbes present in bioflocs showed significantly higher relative abundance in the LW fish gut than the Ctrl fish gut (Fig. S4). This suggested that in-situ biofloc acts as a microbial source and modulates the gut microbiota assembly of fish cultured in the system, which might be potentially beneficial to the growth of Nile tilapia. To be noticed, *Cetobacterium*, Peptostreptococcaceae, and *Terrimicrobium* are all related with fermentative metabolism of peptides and carbohydrates [53–56]. Besides, *Alsobacter* and a genus belonging to the Fusobacteriales were identified as core taxa in LW but were absent in the Ctrl treatment. A species from *Alsobacter* isolated from soil samples could accumulate PHB granules [57]. The members of Fusobacteriales can ferment carbohydrates, amino acids and peptides to produce various short-chain fatty acids [58]. In summary, tilapia cultured in in-situ biofloc system (LW) showed a reduction in abundance of dominant *Cetobacterium,* meanwhile showed higher abundance of genera potentially involved in carbohydrate fermentation and short-chain fatty acids production. In contrast to dietary supplementation of biofloc, in-situ live biofloc changed the gut microbiota that may relate with feed digestion and in part explained the improved growth of fish in the LW treatment.

## Conclusions

Nile tilapia exposed to in-situ biofloc (LW) had a consistently distinct gut microbiota by creating a different microbial community in the water column and the aggravation of biofloc. Rearing tilapia in LW increased the gut microbial diversity by reducing the relative abundance of dominant taxa and increasing the relative abundance of potentially beneficial bacteria. Nile tilapia grew better in LW than tilapia fed with processed live or dead biofloc and tilapia without biofloc. Dietary supplementation of live or dead biofloc at 5 and 10% weight base changed the original microbial composition of biofloc, which did not result in better growth nor influence the gut microbiota of tilapia.

## Supplementary Information

Below is the link to the electronic supplementary material.

Supplementary file1 (DOCX 648 kb)

## Data Availability

The datasets generated and analysed during the current study are available from the corresponding author on reasonable request.

## Code Availability

No special code was developed for data analysis in relation to this research.
