# Supplementary material for: In-Situ Biofloc Affects the Core Prokaryotes Community Composition in Gut and Enhances Growth of Nile Tilapia (Oreochromis niloticus)
Source: Microb Ecol. 2021 Oct 5;84(3):879–92. doi: 10.1007/s00248-021-01880-y (PMC9622544; doi:10.1007/s00248-021-01880-y)
Supplement: Supplementary file 1 — Supplementary file1 (DOCX 648 kb) [file 248_2021_1880_MOESM1_ESM.docx]

# Supplementary material

# *In-situ* biofloc affects the core prokaryotes community composition in gut and enhances growth of Nile tilapia (*Oreochromis niloticus*)

Yale Deng^a^, Klaudyna Borewicz^b,c^, Joost van Loo^a^, Marko Zabala Olabarrieta^b^, Fotini Kokou^a^, Detmer Sipkema^b^, Marc C.J. Verdegem^a­^

*^a^Aquaculture and Fisheries Group, Wageningen University and Research, Wageningen, The Netherlands*

*^b^Laboratory of Microbiology, Wageningen University and Research, Wageningen, The Netherlands*

*^c^Trouw Nutrition R&D, Amersfoort 3811 MH, The Netherlands*

**Correspondence**

*Marc Verdegem, Aquaculture and Fisheries Group, Wageningen University and Research, The Netherlands; Email address: [marc.verdegem@wur.nl](mailto:marc.verdegem@wur.nl)

**Table S1** Comparison of alpha diversity indexes showing statistically significant differences (FDR < 0.05) between different groups. The comparisons were performed by (1) all groups were compared to initial (d0), (2) groups were compared with each other within each timepoint, and (3) the same group was compared across timepoint between d26 and 49. Only statistically significant (FDR < 0.05) comparisons are listed. (a) Shannon index, (b) PD whole tree index, (c) Chao1 index

(a) Shannon index

| **Group1** | **Group2** | **Group1 mean** | **Group1 std** | **Group2 mean** | **Group2 std** | **t stat** | **FDR** |
| --- | --- | --- | --- | --- | --- | --- | --- |
| d0 | d26_LF10 | 3.267 | 0.562 | 4.315 | 0.972 | 2.827 | 0.031 |
| d0 | d26_LW | 3.267 | 0.562 | 4.975 | 0.206 | 10.189 | 0.013 |
| d0 | d49_DF10 | 3.267 | 0.562 | 2.400 | 0.869 | 2.560 | 0.047 |
| d0 | d49_LW | 3.267 | 0.562 | 4.073 | 0.575 | -3.209 | 0.018 |
| d26_LW | d26_Ctrl | 4.975 | 0.206 | 3.883 | 1.127 | 3.550 | 0.010 |
| d26_LW | d26_DF5 | 4.975 | 0.206 | 3.955 | 1.291 | -2.915 | 0.030 |
| d26_LW | d26_DF10 | 4.975 | 0.206 | 2.822 | 1.226 | 6.479 | 0.016 |
| d26_LW | d26_LF10 | 4.975 | 0.206 | 4.315 | 0.972 | 2.486 | 0.046 |
| d26_LF5 | d26_DF10 | 4.358 | 1.298 | 2.822 | 1.226 | 3.099 | 0.016 |
| d26_LF10 | d26_DF10 | 4.315 | 0.972 | 2.822 | 1.226 | 3.570 | 0.007 |
| d49_LW | d49_Ctrl | 4.073 | 0.575 | 2.984 | 1.048 | 3.376 | 0.011 |
| d49_LW | d49_DF10 | 4.073 | 0.575 | 2.400 | 0.869 | 6.003 | 0.010 |
| d49_LW | d49_LF5 | 4.073 | 0.575 | 2.697 | 1.010 | 4.429 | 0.009 |
| d49_LW | d49_LF10 | 4.073 | 0.575 | 1.173 | 4.073 | -3.355 | 0.008 |
| d26_LW | d49_LW | 4.975 | 0.206 | 4.073 | 0.575 | 5.524 | 0.006 |
| d26_LF5 | d49_LF5 | 4.358 | 1.298 | 2.697 | 1.010 | -3.664 | 0.009 |
| d26_LF10 | d49_LF10 | 4.315 | 0.972 | 2.910 | 1.173 | -3.500 | 0.039 |

(b) PD whole tree index

| **Group1** | **Group2** | **Group1 mean** | **Group1 std** | **Group2 mean** | **Group2 std** | **t stat** | **FDR** |
| --- | --- | --- | --- | --- | --- | --- | --- |
| d0 | d26_Ctrl | 7.982 | 2.388 | 10.772 | 2.077 | -2.776 | 0.031 |
| d0 | d26_LF5 | 7.982 | 2.388 | 12.108 | 3.212 | -3.125 | 0.016 |
| d0 | d26_LF10 | 7.982 | 2.388 | 11.670 | 1.892 | 4.004 | 0.011 |
| d0 | d26_DF5 | 7.982 | 2.388 | 11.785 | 2.947 | 3.102 | 0.030 |
| d0 | d26_LW | 7.982 | 2.388 | 13.227 | 0.580 | 7.772 | 0.020 |
| d0 | d49_LW | 7.982 | 2.388 | 10.573 | 1.202 | -3.374 | 0.022 |
| d26_LW | d26_Ctrl | 13.227 | 0.580 | 10.772 | 2.077 | 4.225 | 0.010 |
| d26_LW | d26_DF10 | 13.227 | 0.580 | 9.714 | 4.042 | 3.219 | 0.016 |
| d26_LW | d26_LF10 | 13.227 | 0.580 | 11.670 | 1.892 | 2.945 | 0.018 |
| d49_LW | d49_DF10 | 10.573 | 1.202 | 7.903 | 2.196 | 3.991 | 0.013 |
| d49_DF5 | d49_DF10 | 10.975 | 2.955 | 7.903 | 2.196 | 3.079 | 0.021 |
| d26_LW | d49_LW | 13.227 | 0.580 | 10.573 | 1.202 | 7.439 | 0.009 |
| d26_LF5 | d49_LF5 | 12.108 | 3.212 | 8.820 | 2.456 | -2.953 | 0.025 |
| d26_LF10 | d49_LF10 | 11.670 | 1.892 | 8.476 | 3.099 | -3.323 | 0.023 |

(c) Chao1 index

| **Group1** | **Group2** | **Group1 mean** | **Group1 std** | **Group2 mean** | **Group2 std** | **t stat** | **FDR** |
| --- | --- | --- | --- | --- | --- | --- | --- |
| d0 | d26_LF5 | 54.351 | 19.426 | 91.367 | 37.871 | -2.571 | 0.047 |
| d0 | d26_LW | 54.351 | 19.426 | 97.974 | 7.472 | 7.458 | 0.007 |
| d26_LW | d26_DF10 | 97.974 | 7.472 | 57.486 | 44.186 | 3.381 | 0.009 |
| d26_LW | d26_Ctrl | 97.974 | 7.472 | 73.034 | 25.943 | 3.428 | 0.021 |
| d26_LF10 | d26_DF10 | 91.558 | 24.628 | 57.486 | 44.186 | 2.520 | 0.033 |
| d26_LW | d49_LW | 97.974 | 7.472 | 67.172 | 15.956 | 6.541 | 0.010 |
| d26_LF5 | d49_LF5 | 91.367 | 37.871 | 50.970 | 29.137 | -3.069 | 0.042 |
| d26_LF10 | d49_LF10 | 91.558 | 24.628 | 48.065 | 31.000 | -4.166 | 0.039 |
| d49_LW | d49_DF10 | 67.172 | 15.956 | 40.667 | 25.038 | 3.340 | 0.017 |

**Table S2** ANOSIM comparison of weighted Unifrac distances between treatment groups

| **Group 1** | **Group 2** | **t statistic** | **Parametric p-value** | **Nonparametric p-value** |
| --- | --- | --- | --- | --- |
| d26_Ctr | d26_LW | -9.9794479 | 5.79E-19 | **0.001** |
| d26_Ctr | d26_LF | -2.7143777 | 0.0068929 | 0.005 |
| d26_Ctr | d26_DF | 2.089983 | 0.0371428 | 0.029 |
| d26_LW | d26_LF | -12.769674 | 2.32E-32 | 0.001 |
| d26_LW | d26_DF | -6.9700624 | 9.86E-12 | 0.001 |
| d26_LF | d26_DF | 8.2229159 | 8.88E-16 | 0.001 |
| d49_Ctr | d49_DF | 2.5098727 | 0.0123955 | 0.014 |
| d49_LW | d49_DF | 3.1405303 | 0.0017845 | 0.002 |
| d49_LW | d49_DF | 3.1405303 | 0.0017845 | 0.002 |
| d49_Ctr | d49_LW | -0.5429835 | 0.0017644 | 0.001 |

**Table S3** Taxonomy of significantly different abundant taxa between different treatment groups on both d26 and d49

| **Taxonomy** | **Classification** |
| --- | --- |
| *g_Cetobacterium* | k_Bacteria;p_Fusobacteria;c_Fusobacteriia;o_Fusobacteriales;f_Fusobacteriaceae;g_Cetobacterium |
| *g_Terrimicrobium* | k_Bacteria;p_Verrucomicrobia;c_Spartobacteria;o_Chthoniobacterales;f_Chthoniobacterales_Incertae_Sedis;g_Terrimicrobium |
| *g_Mycobacterium* | k_Bacteria;p_Actinobacteria;c_Actinobacteria;o_Corynebacteriales;f_Mycobacteriaceae;g_Mycobacterium |
| *f_Planctomycetaceae_g_uncultured* | k_Bacteria;p_Planctomycetes;c_Planctomycetacia;o_Planctomycetales;f_Planctomycetaceae;g_uncultured |
| *f_Planctomycetaceae_g* | k_Bacteria;p_Planctomycetes;c_Planctomycetacia;o_Planctomycetales;f_Planctomycetaceae;g_ |
| *f_MNG7_g_uncultured_bacterium* | k_Bacteria;p_Proteobacteria;c_Alphaproteobacteria;o_Rhizobiales;f_MNG7;g_uncultured_bacterium |
| *p_Planctomycetes_g* | k_Bacteria;p_Planctomycetes;c_;o_;f_;g_ |
| *o_Burkholderiales_g* | k_Bacteria;p_Proteobacteria;c_Betaproteobacteria;o_Burkholderiales;f_;g_ |
| *g_Variibacter* | k_Bacteria;p_Proteobacteria;c_Alphaproteobacteria;o_Rhizobiales;f_Xanthobacteraceae;g_Variibacter |
| *g_Pir4_lineage* | k_Bacteria;p_Planctomycetes;c_Planctomycetacia;o_Planctomycetales;f_Planctomycetaceae;g_Pir4_lineage |
| *o_JG30_KF_CM45_g* | k_Bacteria;p_Chloroflexi;c_Thermomicrobia;o_JG30-KF-CM45;f_uncultured_bacterium;g_<empty> |
| *o_Chlamydiales_g* | k_Bacteria;p_Chlamydiae;c_Chlamydiae;o_Chlamydiales;f_;g_ |
| *f_Microbacteriaceae_g* | k_Bacteria;p_Actinobacteria;c_Actinobacteria;o_Micrococcales;f_Microbacteriaceae;g_ |
| *g_Nordella* | k_Bacteria;p_Proteobacteria;c_Alphaproteobacteria;o_Rhizobiales;f_Rhizobiales_Incertae_Sedis;g_Nordella |
| *f_Rhodobacteraceae_g* | k_Bacteria;p_Proteobacteria;c_Alphaproteobacteria;o_Rhodobacterales;f_Rhodobacteraceae;g_ |
| *Archaea_g* | k_Archaea;p_Thaumarchaeota;c_Soil_Crenarchaeotic_Group(SCG);o_;f_;g_ |
| *f_Porphyromonadaceae_g_uncultured* | k_Bacteria;p_Bacteroidetes;c_Bacteroidia;o_Bacteroidales;f_Porphyromonadaceae;g_uncultured |
| *g_Alsobacter* | k_Bacteria;p_Proteobacteria;c_Alphaproteobacteria;o_Rhizobiales;f_Rhizobiales_Incertae_Sedis;g_Alsobacter |
| *g_Reyranella* | k_Bacteria;p_Proteobacteria;c_Alphaproteobacteria;o_Rhodospirillales;f_Rhodospirillales_Incertae_Sedis;g_Reyranella |
| *g_Candidatus_Microthrix* | k_Bacteria;p_Actinobacteria;c_Acidimicrobiia;o_Acidimicrobiales;f_Acidimicrobiales_Incertae_Sedis;g_Candidatus_Microthrix |
| *f_Geodermatophilaceae_g_uncultured* | k_Bacteria;p_Actinobacteria;c_Actinobacteria;o_Frankiales;f_Geodermatophilaceae;g_uncultured |
| *g_Meganema* | k_Bacteria;p_Proteobacteria;c_Alphaproteobacteria;o_Rhizobiales;f_Methylobacteriaceae;g_Meganema |
| *g_Iamia* | k_Bacteria;p_Actinobacteria;c_Acidimicrobiia;o_Acidimicrobiales;f_Iamiaceae;g_Iamia |
| *g_Macellibacteroides* | k_Bacteria;p_Bacteroidetes;c_Bacteroidia;o_Bacteroidales;f_Porphyromonadaceae;g_Macellibacteroides |
| *g_Deefgea* | k_Bacteria;p_Proteobacteria;c_Betaproteobacteria;o_Neisseriales;f_Neisseriaceae;g_Deefgea |
| *g_Sorangium* | k_Bacteria;p_Proteobacteria;c_Deltaproteobacteria;o_Myxococcales;f_Polyangiaceae;g_Sorangium |
| *p_TM6_Dependentiae_g* | k_Bacteria;p_TM6_(Dependentiae);c_;o_;f_;g_ |
| *o_Fusobacteriales_g* | k_Bacteria;p_Fusobacteria;c_Fusobacteriia;o_Fusobacteriales;f_;g_ |
| *f_TM146_g_uncultured_bacterium* | k_Bacteria;p_Actinobacteria;c_Thermoleophilia;o_Solirubrobacterales;f_TM146;g_uncultured_bacterium |
| *p_TM6_Dependentiae_uncultured_g* | k_Bacteria;p_TM6_(Dependentiae);c_uncultured_bacterium;o_<empty>;f_<empty>;g_<empty> |
| *f_Holosporaceae_g_uncultured* | k_Bacteria;p_Proteobacteria;c_Alphaproteobacteria;o_Rickettsiales;f_Holosporaceae;g_uncultured |
| *o_Bacillales_g_uncultured_bacterium* | k_Bacteria;p_Firmicutes;c_Bacilli;o_Bacillales;f_uncultured;g_uncultured_bacterium |
| *f_Rickettsiaceae_g* | k_Bacteria;p_Proteobacteria;c_Alphaproteobacteria;o_Rickettsiales;f_Rickettsiaceae;g_ |
| *c_SBR2076_g* | k_Bacteria;p_Chloroflexi;c_SBR2076;o_uncultured_bacterium;f_<empty>;g_ |

**Table S4** Genus level taxa showing significantly (FDR < 0.05) difference in relative abundant in LW vs. Ctrl, but not in LW vs. LF comparison

| **Time** | **Taxonomy** | **Average relative abundance** | | |  | **FDR_P** | |
| --- | --- | --- | --- | --- | --- | --- | --- |
|  |  | **Ctrl** | **LW** | **LF** |  | **LW vs Ctrl** | **LW vs LF** |
| d26 | *f_Brevinemataceae_g* | 0.015 | 0.0007 | 0.0037 |  | 0.041 | 0.193 |
|  | *Cetobacterium* | 0.4394 | 0.1278 | 0.314 |  | 0.018 | 0.483 |
|  | *Cupriavidus* | 0.0027 | 0.0005 | 0.0028 |  | 0.045 | 0.133 |
|  | *Iamia* | 0.0001 | 0.0014 | 0.0011 |  | 0.002 | 0.665 |
| d49 | *f_Peptostreptococcaceae_g* | 0.0105 | 0.0279 | 0.0235 |  | 0.024 | 0.245 |
|  | *Turicibacter* | 0.0002 | 0.0014 | 0.0008 |  | 0.043 | 0.201 |
|  | *o_Rhizobiales_g* | 0.0007 | 0.0037 | 0.0023 |  | 0.043 | 0.273 |

**Table S5** ANOSIM comparison between different (a) filtered tank water samples and (b) feed samples based on Bray-Curtis distances

| **Time** | **Group 1** | **Group 2** | **R statistic** | ***P*-value** |
| --- | --- | --- | --- | --- |
| d0 | LW | Ctrl | 0.519 | 0.100 |
|  | LW | LF | 0.531 | 0.012 |
|  | LW | DF | 1 | 0.012 |
|  | Ctrl | LF | 0.136 | 0.202 |
|  | Ctrl | DF | 0.228 | 0.167 |
|  | LF | DF | -0.078 | 0.911 |
| d26 | LW | Ctrl | 0.778 | 0.001 |
|  | LW | LF | 0.998 | 0.001 |
|  | LW | DF | 1 | 0.001 |
|  | Ctrl | LF | 0.077 | 0.176 |
|  | Ctrl | DF | 0.155 | 0.056 |
|  | LF | DF | 0.261 | 0.001 |
| d49 | LW | Ctrl | 0.465 | 0.001 |
|  | LW | LF | 0.321 | 0.001 |
|  | LW | DF | 0.392 | 0.001 |
|  | Ctrl | LF | 0.012 | 0.363 |
|  | Ctrl | DF | 0.038 | 0.240 |
|  | LF | DF | 0.019 | 0.095 |

(a) filtered tank water samples

(b) feed samples

| **Time** | **Group 1** | **Group 2** | **R statistic** | ***P*-value** |
| --- | --- | --- | --- | --- |
| d0 | bioflocs | DF | 0.867 | 0.002 |
|  | bioflocs | LF | 0.864 | 0.002 |
|  | bioflocs | LW/Ctrl | 0.908 | 0.018 |
|  | DF | LF | -0.019 | 0.554 |
|  | DF | LW/Ctrl | 0.926 | 0.012 |
|  | LF | LW/Ctrl | 0.926 | 0.012 |
| d26 | bioflocs | DF | 1 | 0.002 |
|  | bioflocs | LF | 1 | 0.002 |
|  | bioflocs | LW/Ctrl | 1 | 0.048 |
|  | DF | LF | -0.059 | 0.662 |
|  | DF | LW/Ctrl | 1 | 0.036 |
|  | LF | LW/Ctrl | 1 | 0.036 |
| d49 | bioflocs | DF | 0.848 | 0.002 |
|  | bioflocs | LF | 0.88 | 0.002 |
|  | bioflocs | LW/Ctrl | 1 | 0.018 |
|  | DF | LF | 0.143 | 0.1 |
|  | DF | LW/Ctrl | 1 | 0.012 |
|  | LF | LW/Ctrl | 1 | 0.012 |


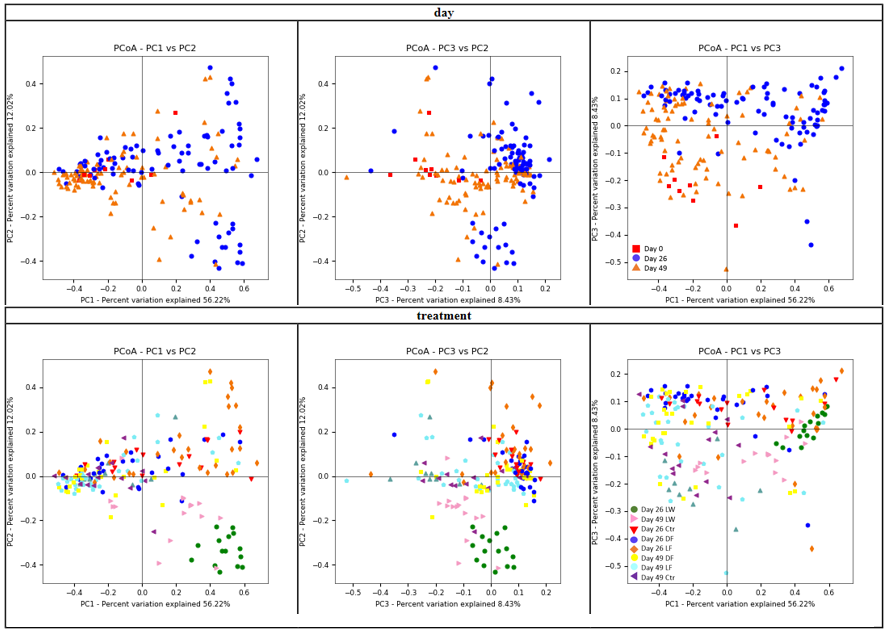


**Figure S1** PCoA showing spatial distribution of gut prokaryotic communities based on weighted UniFrac distances. The color of samples was coded according to sampling timepoints (top panel) or treatment groups (bottom panel). The green ellipse indicates samples from the LW treatment.


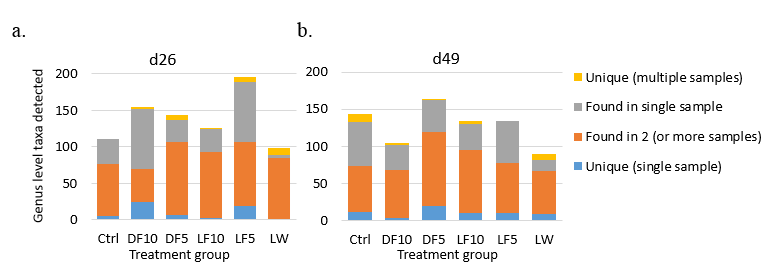


**Figure S2** Prevalence of genus level taxa in each treatment at (a) d26 and (b) d49. Unique (single sample) - indicates these genera were detected only in one treatment and were present in only one sample in that treatment; Unique (multiple samples) - indicates these genera were detected only in one treatment and were present in more than 2 samples in that treatment; Found in single sample - indicates those genera were only detected in one sample of that treatment but were not unique to that treatment; Found in 2 (or more samples) - indicates those genera were detected in more than one samples of that treatment and were not unique to one treatment.


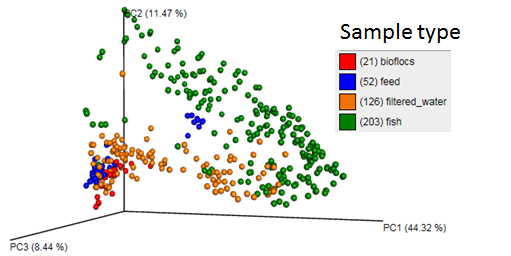


**Figure S3** PCoA showing spatial distribution of bioflocs, feed, filtered tank water and fish gut prokaryotic communities based on weighted UniFrac distances.


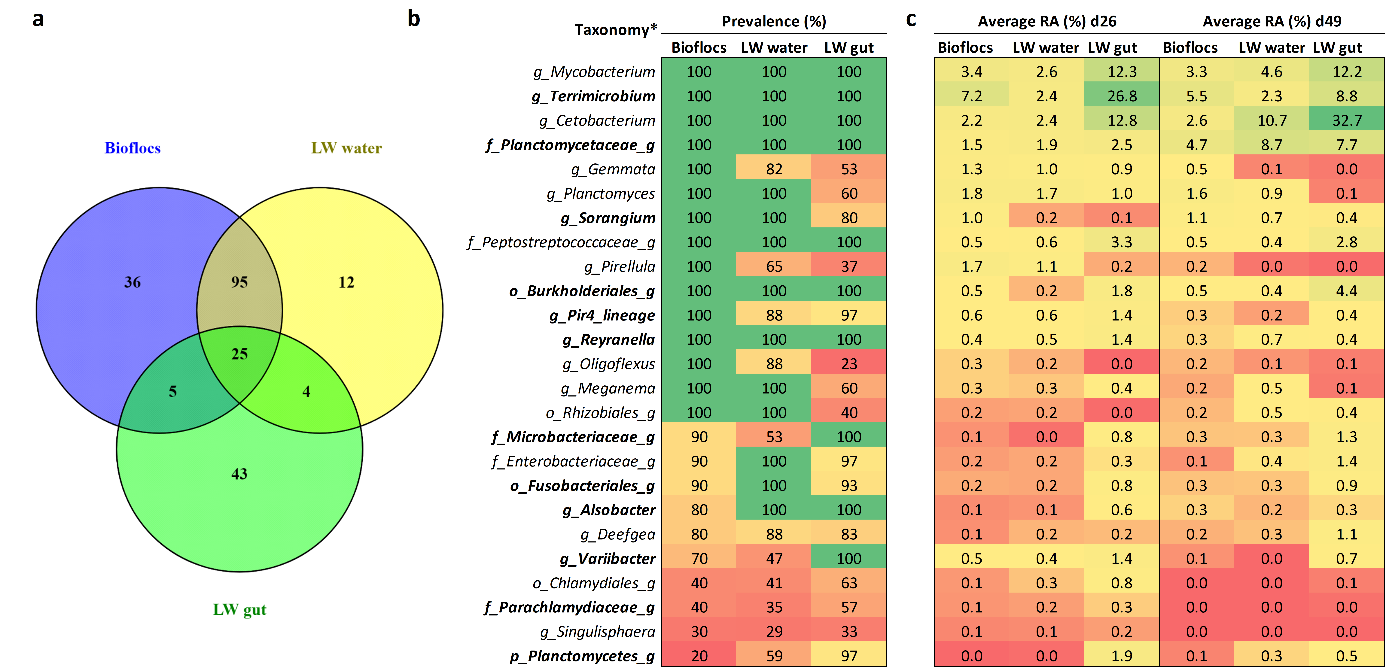


**Figure S4** (a) Venn diagram showing the taxa shared between bioflocs, LW filtered water and LW gut samples, 25 taxa were shared in bioflocs, LW water and LW gut samples. (b) Prevalence (%) and (c) average relative abundance of the 25 shared taxa in bioflocs, LW water and LW gut samples on d26 and d49. *The taxonomy had significantly higher relative abundance in LW gut than Ctrl gut are showed in bolded text.
